# Transporting cells over several days without dry-ice

**DOI:** 10.1242/jcs.238139

**Published:** 2019-11-01

**Authors:** Sally P. Wheatley, Denys N. Wheatley

**Affiliations:** 1 School of Life Sciences, University of Nottingham, Queen's Medical Centre, Nottingham NG7 2UH, UK; 2 BioMedES, AB51 0LX, UK

**Keywords:** Cell culture, LMT agarose, Cell shipment

## Abstract

This paper describes a simple, hazard-free and inexpensive procedure that allows researchers to send cultured cells across the globe at ambient temperatures. The method enables transit of up to 2 weeks without compromising cell recovery. Its use will assist collaborators in distant laboratories to exchange cells without using dry-ice.

## Abstract (precis)

**Summary:** An easy dry-ice-free method of sending cultured cells across the globe at ambient temperatures.

## INTRODUCTION

For many decades, the conventional method of sending cells from A to B globally has been as frozen samples dispatched by courier in several kilograms of dry-ice. From storage to delivery, the cost is high and the procedure hazardous, with many couriers refusing to take cargo on dry-ice. Delay in transit is common (e.g. slow customs inspection), and when this occurs the dry-ice often evaporates, leaving the cells bathed in cryoprotectant (10% DMSO), which is cytotoxic at ambient temperatures. While cell culture facilities will most likely continue to use this mode of shipping because their collections are held in liquid nitrogen, other methods are becoming available that minimise these frustrations and maximise cell recovery post transit. They also have the added advantage of eliminating the large CO_2_ emission associated with using dry-ice. Some researchers simply send culture flasks of cells filled with medium and tightly secured caps, but here agitation can cause cell monolayers to detach; however, more importantly, liquids pose a spill hazard, and are prohibited in the mail and air travel. To circumvent these issues, we and others have turned to gel-based medium to transport biological samples, as published in a broad patent in 2002 (Baust et al., 2002), and found in Bagasra et al. (1985), Brendel et al. (1994) and Stevens et al. (2005). Undoubtedly there is a need for a simple, inexpensive and non-hazardous method of transporting cell cultures between collaborators, particularly in this era in which designer cell lines are becoming centre stage. What follows is a brief description of a tried and tested method we have devised that can fulfil this need.

## RESULTS AND DISCUSSION

**Fig. 1.**
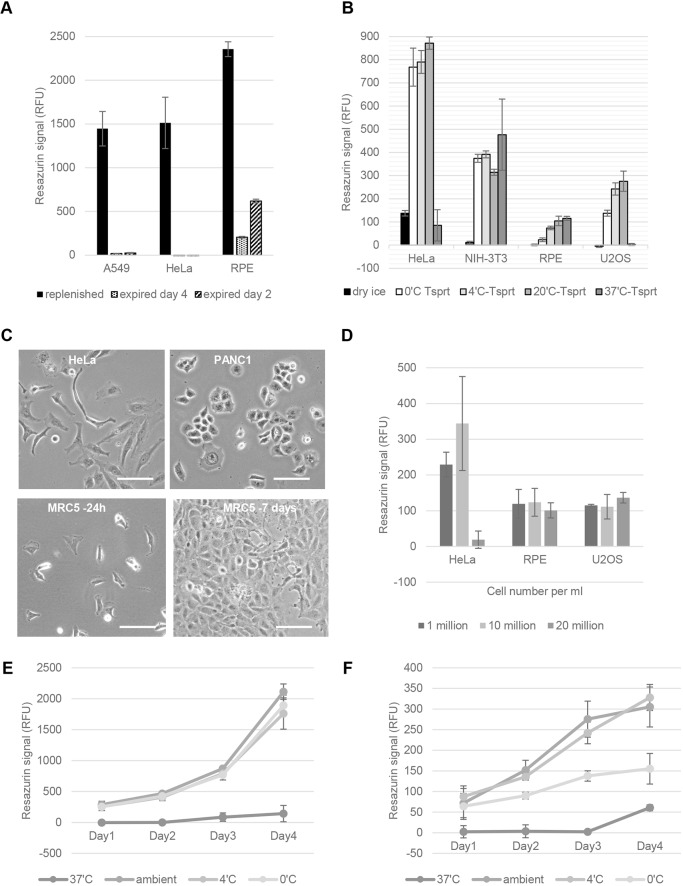
**Cells can be transported without dry ice.** (A) The cell lines indicated were incubated on dry-ice in 90% FCS with 10% DMSO for 7 days and cell viability (expressed in relative fluorescence units, RFU) was assessed using the resazurin assay. (B) The cell lines (5×10^6^/ml) indicated were incubated in FCS in DMSO and held on dry-ice or resuspended in Transporter (Tsprt) and incubated at different temperatures before viability was assessed, as in A. (C) Phase-contrast images of recovering cells after 7 days in Transporter and seeded 72 h before imaging (HeLa and PANC2), or 96 h in Transporter and imaged 24 h post seeding (MRC5). Scale bars: 100 µm. (D) Cells were transported at three different densities at ambient temperature and cell viability assessed 24 h post-seeding. Graphs show mean±s.d within a single experiment representative of *n*=3 independent experiments. (E,F) Growth of HeLa (E) and U2OS (F) cells over 4 days after being kept in Transporter for 3 days at the indicated temperatures. All graphs show representative experiments performed at least three independent times. Internal repeats were carried out in quadruplicate, bars/points indicated the mean, and error bars show s.d. within the representative experiment.

First, to demonstrate the risk of sending cells on dry-ice, three cell lines were resuspended in 90% fetal calf serum (FCS) with 10% DMSO and held on dry-ice for 7 days. For one vial of each line, the dry-ice was regularly replenished, but for the other samples it evaporated completely within ∼4 days. Cell viability was assessed 72 h post-seeding, using the metabolic resazurin-based assay. Recovery from frozen was successful when the dry-ice was maintained throughout transit, but recovery was poor (RPE) or non-existent (U2OS and HeLa) when the dry-ice had expired (Fig. 1A).

We have compared the efficacy of recovery between cells held on dry-ice and those in our ‘Transporter’. Four cell lines were resuspended in either 90% FCS with 10% DMSO and placed on dry-ice (replenished) or in Transporter held at constant temperatures of 0°C (ice), 4°C, ∼20°C (ambient) or 37°C. After 72 h incubation, cell viability was assessed using resazurin. All cell lines recovered better in Transporter than on dry-ice when previously kept at 0°C, 4°C or 20°C (Fig. 1B), which is also evident from their morphology when reattached and growing in Petri dishes (Fig. 1C). Recovery after 72 h transit at 37°C in Transporter was cell line dependent, with untransformed NIH 3T3 (mouse fibroblasts) and RPE cells (human retinal epithelial) recovering successfully (Fig. 1B). For transformed cell lines (e.g. HeLa and A549), transportation for 7 days up to 27°C was well tolerated, but temperatures of 32°C and above were not. The most robust cells tolerated up to 3 weeks of transport (e.g. U2OS and MRC5) at ambient temperature (20–22°C). A summary of similar findings using eight different cell lines kept for different lengths of time in Transporter is shown in Table 1. [However, not all cell lines will necessarily respond in the exactly the same way, and thus it is worth checking carefully beforehand their ability to endure the conditions of transportation.]

**Table 1.**
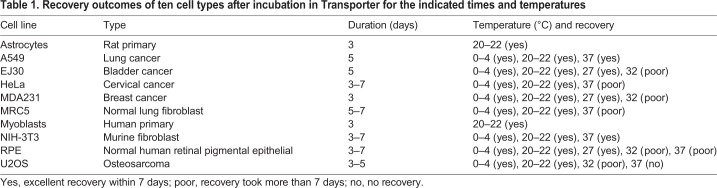
**Recovery outcomes of ten cell types after incubation in Transporter for the indicated times and temperatures**

Further experiments were carried out to determine the optimal cell density and maximum duration tolerated in transit. To determine the optimum cell density, we assessed cell viability post-transit for three cell lines transported for 72 h at ambient temperature at 10^6^, 10×10^6^ or 20×10^6^ cells per ml. Notably, although RPE and U2OS cells fared similarly at all densities, HeLa cells did not survive well at 20×10^6^ cells/ml (Fig. 1D). When transit was carried out at 4°C, all three lines did less well at 10^6^ cells/ml (data not shown); therefore subsequent experiments were carried out with cells at between 10^6^ and 10^7^ cells/ml, with the preferred density being 5×10^6^ cells/ml. Growth curves for HeLa and U2OS cells recovering after 3 days storage in Transporter at different temperatures are shown in Fig 1E,F. When cell viability was compared between cells transported for 72 h versus 240 h, for all four cell lines tested there was a decline in cell viability (Fig. S1B), indicating that shorter transit is better and providing some evidence that these cells do not proliferate when transported at ambient temperature.

**Fig. 2.**
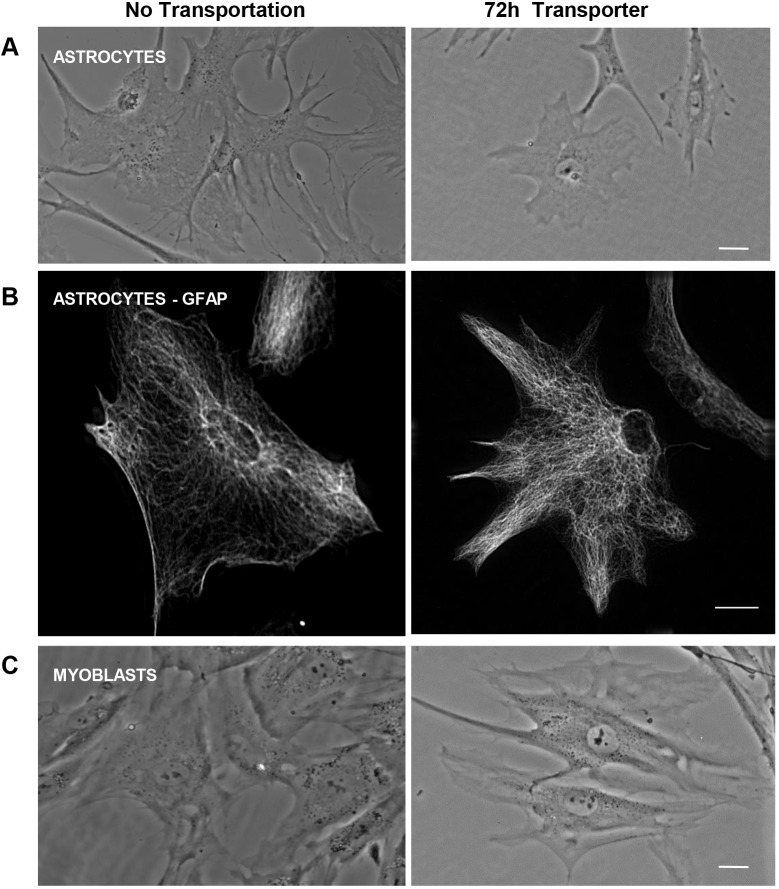
**Primary cells can tolerate 72 h transit in Transporter.** (A,B) Rat astrocytes were either trypsinised and seeded (no transportation) or incubated in Transporter for 72 h then seeded, and imaged in phase contrast (A) 24 h later or (B) to prove that they retained their astrocytic characteristics, they were fixed and immunostained for GFAP and imaged using a fluorescence microscope. (C) Human myoblasts were treated in the same way as rat astrocytes in A. Scale bars: 20 µm.

Having established that immortalised cell lines could tolerate transportation in this way, we next asked whether primary cells would also survive. Rat astrocytes (Fig. 2A,B) and human myoblasts (Fig. 2C), were seeded 24 h prior to imaging either directly from the stock flasks (no transportation) or after 72 h in Transporter. Phase imaging demonstrated that recovery was excellent in each case and cells looked similar regardless of whether they had been transported or simply passaged. Moreover, to prove that astrocytes retained their normal and defining characteristics after transport, we immunostained them with anti-GFAP antibodies and observed an extensive filamentous network (Fig. 2B). Thus, Transporter can also be used for primary cell transit, at least for up to 72 h.

Given that organisms, such as zebrafish (Percival and Parant, 2016), organoids and eggs (Udan et al., 2014) are routinely immobilised in agarose for analysis by live fluorescence confocal and light sheet imaging, it is unsurprising that this method is so effective. Indeed, our Transporter medium is similar in many respects to the natural gel of amphibian eggs, which supports development to the juvenile stage of the animal. Our Transporter has successfully supported the carriage of developing *Drosophila* (data not shown). Thus, it will be interesting to determine whether this method can aid the transit of germ cells (Hatakeyama et al., 2017) – which would aid breeding programmes – and stem cells, which would be medically beneficial, as also to others, such as veterinarians. Should Transporter support the preservation of small pieces of tissues (biopsies and organs), this would have huge medical implications, with the most immediate benefactors being transplant patients. And finally, Transporter could also be adopted to improve containment of species presenting biological safety hazards that need to be shipped across the globe for diagnostic purposes.

### Conclusion

We have disclosed a simple, inexpensive and non-hazardous method for sending cultured cells between laboratories. While there is some variation in survival depending upon cell type, excellent recovery is seen when cells are transported at 5×10^6^ cells/ml within the temperature range 4–27°C for 7 days, and where tested there have been cases of very good recovery after 3 weeks at ambient temperature (20–22°C). For the best results, we recommend sending cells in small aliquots of Transporter (100 µl) in microfuge tubes at 5×10^6^ cells/ml, ensuring that the gel pellet is dispersed as fully as possible prior to seeding.

Proof of principle was established on three occasions, with cells being taken as hand-luggage in transit (for up to 5 days) from the UK to Hong Kong, where excellent, fast recovery was observed after seeding. The method has since been successfully trialled by at least seven independent colleagues.

## MATERIALS AND METHODS

### Cell culture

All cells (having been tested as mycoplasma free) were cultured in DMEM with 10% fetal calf serum (FCS), supplemented with 1% glutamine and 1% antibiotic and antimycotic, herein referred to as ‘1× complete DMEM’. Cells were maintained at 37°C in a humid incubator with 5% CO_2_ in air. Prior to transit, cells were harvested by trypsinisation, pelleted by centrifugation (500 ***g*** for 3 min), and resuspended at 0.5×10^7^–1×10^7^ cells/ml in 1× complete DMEM. Rat astrocytes were isolated (ethical approval AWERB ref 000148) and cultured in DMEM supplemented with 500 μg/ml proline as described previously (James et al., 2011). Human myoblasts were isolated with University of Nottingham ethical approval (G11092014SoLS) and cultured in F10/HAMS with 20% FCS (O'Leary et al., 2018). Cell culture reagents were supplied by Thermo Fisher Scientific, unless stated otherwise. The following cell lines have been used, although not all will be referred to in this report: normal lines, RPE-1, human retinal pigmented epithelial; 3T3, murine fibroblast; MRC5, human lung fibroblasts; and HEK293: human embryonic kidney; malignant (human) cell lines, HeLa: cervical; U2OS, osteosarcoma; Hct116, colorectal; MDA-MB231, breast; SKBR3, breast; EJ30, bladder; and MCF7, breast.

### Primary cell lines

Rat astrocytes were a gift from Andrew Bennett, SoLS, Nottingham, UK; human myoblasts (O’Leary et al., 2018) were a gift from Kostas Tsintzas, SoLS, Nottingham, UK.

### Preparation of ‘Transporter’

A 2% solution of low-melting temperature (LMT) agarose (Sigma-Aldrich catalogue number A9414) was prepared in PBS, sterilised by autoclaving, cooled and supplemented with filter-sterilised HEPES at 20 mM (pH 7.4), then held at 37°C ready for the addition of cells. Reagents were supplied by Sigma-Aldrich.

### Cells in ‘Transporter’

Cells at 1×10^7^ cells/ml resuspended in complete DMEM were mixed 1:1 with pre-warmed Transporter, pipetted into 1.5 ml microfuge tubes in ∼100 µl aliquots, and sealed with parafilm (Fig. S1C). When using PBS as the diluent for LMT agarose, 1× complete medium, as normally used for the particular cell type, is recommended. If LMT is constituted in water, then 2× complete medium should be used to ensure the carrier is isotonic; however, this will require medium to be prepared from powdered stock.

The tubes were put into ‘Jiffy’ bags (size A) and sent to their destinations by regular mail, air-mail or courier, with associated documentation.

### Recovery

Upon receipt, the microfuge tubes containing the cells are placed on a heat-block at 37°C, and 1 ml of pre-warmed complete medium was added, and the gel pellet thoroughly dispersed by pipetting intermittently for 5–10 min. The cells were then seeded on to Petri dishes, welled plates, culture flasks or onto live imaging chambers with pre-warmed medium in the usual manner.

### Resazurin assay

To assess cell recovery, cell number was monitored using a standard resazurin assay. Briefly, cells were seeded into 96-well plates in quadruplicate, and incubated for 1 h at 37°C with 10 µg/ml resazurin diluted in complete DMEM before being read on a Fluostar Galaxy spectrophotometer with excitation set at 530 nm and emission to 590 nm. Note that each cell line has a different metabolic profile (Fig. S1A), therefore the heights of the bars should only be compared within the dataset for a single cell type.

### Imaging

#### Phase-contrast imaging

Cell attachment and morphology was followed using a standard inverted Nikon microscope fitted with a ×20 or ×40 objective, and imaged in phase-contrast using a Nikon D3000 camera.

#### Fluorescence imaging

Primary cells, grown in Ibidi live imaging chambers, were washed with pre-warmed PBS, fixed with 4% formaldehyde/PBS (5 min, 37°C), permeabilised with 0.15% Triton X-100 in PBS (2 min, 37°C), blocked with PBS/1%BSA/azide (15 min, RT) and immunostained to visualise the astrocyte specific marker glial fibrillary acid protein (GFAP), with anti-GFAP antibodies, (Cell Signaling Technology, mouse monoclonal GA5; 1:300; 1 h, room temperature), followed by 1:200 fluorescein conjugated anti-mouse-Ig (Vector, 1:200). Images were acquired using an Olympus IX71 microscope fitted with a ×40 (NA 1.3) oil objective, using Deltavision software and a CoolSnap camera. A single deconvolved 0.3 µm optical section is shown. TIFF images were prepared in ImageJ.

### Cell Transporter protocol

#### Sending cells

1. Prepare a 2% solution of low-melting temperature (LMT) agarose (Sigma-Aldrich, cat. no. A9414) in PBS, dissolve and sterilise by autoclaving, then cool to 37°C.
2. Under sterile conditions add HEPES to a final concentration of 20 mM (pH 7.4), mix, aliquot (see additional notes) and store, or use immediately.
3. Harvest exponentially growing adherent cells by trypsinisation and re-suspend at 10^7^ cells/ml in the regular complete medium used for that cell line.
4. Pipette 50 µl sterile LMT agarose into a pre-warmed 1.5 ml microfuge tube.
5. Pipette 50 µl of the cell suspension into the warm LMT solution and mix gently by pipetting (1:1 cell to LMT ratio). Place on rack at room temperature to cool until gel forms (30–60 s).
6. Seal tube with parafilm and place in padded envelope with associated documentation.
7. Send via mail, air-mail or courier, aiming for arrival at destination in preferably less than 1 week.

#### Receiving cells

1. Under sterile conditions, add 1 ml of pre-warmed complete medium to the microfuge tube, pipette to disperse and break the gel pellet.
2. Incubate at 37°C for a further 15–30 min. Re-pipette until the gel has been fully dispersed.
3. Transfer to awaiting tissue culture vessel and culture using normal procedures.

#### Additional notes

1. 2% LMT/HEPES can be stored at 4–22°C for 2 months. *Do not freeze*. LMT can be re-melted by microwaving gently for several seconds or heating to 60°C. Cool to 37°C before use. 1–2 ml aliquots made in 5 ml tubes are most convenient for cell culture.
2. By placing solution on a rack in step 4 (sending), the pellet forms at the bottom of the tube. This minimises evaporation, and, if cells are no longer a discrete pellet at the destination, will suggest that a temperature of >37°C was exceeded during transit.
3. Optimal density may vary with cell type.
4. The procedure can be scaled up, but ensure that there is sufficient space for the addition of 10 volumes of medium upon receipt.
5. Efficiency of recovery improves with greater dispersion of the gel pellet.

## Supplementary Material

Supplementary information
