# Supplementary material for: Transporting cells over several days without dry-ice
Source: J Cell Sci. 2019 Nov 1;132(21):jcs238139. doi: 10.1242/jcs.238139 (PMC6857593; doi:10.1242/jcs.238139)
Supplement: Supplementary information [file joces-132-238139-s1.pdf]

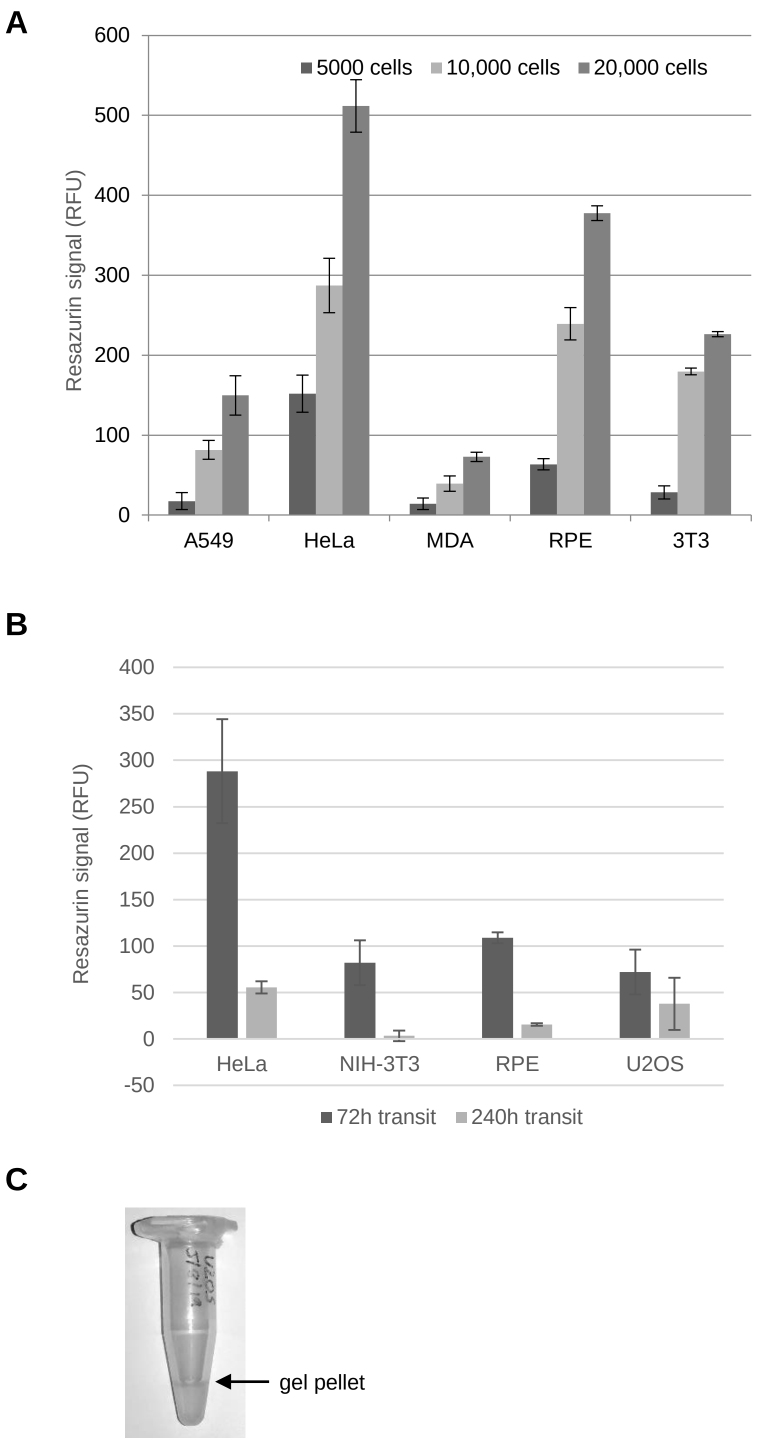

Figure S1. Cell lines have different metabolic profiles and do not proliferate in transit. (A) 5, 10 or 20 x 10^3 cells were seeded in a 96 well plate in triplicate, allowed to adhere for 4h, then a rezasurin assay carried out. **(B)** Cells were seeded after 72h or 240h in transit, allowed 24h to recover then viability assessed. Cell number diminished with increased transit time suggesting loss of viability and no proliferation. **(C)** Microfuge tube containing 100 µl cells in Transporter ready for transit.

Journal of Cell Science • Supplementary information
